# Supplementary material for: Insulin-Loaded Barium Salt Particles Facilitate Oral Delivery of Insulin in Diabetic Rats
Source: Pharmaceutics. 2020 Jul 29;12(8):710. doi: 10.3390/pharmaceutics12080710 (PMC7464671; doi:10.3390/pharmaceutics12080710)
Supplement: Supplementary file 1 [file pharmaceutics-12-00710-s001.pdf]

pharmaceutics

# Supplementary Materials: Insulin-Loaded Barium Salt Particles Facilitate Oral Delivery Of Insulin In Diabetic Rats

**Zaman R, Karim ME, Othman I, Zaini A and Chowdhury EH**

## Concentration vs. Absorbance Standard Curve Prepared with Free FITC-Insulin Samples

Figure S1: Standard curve was prepared by diluting free FITC-Insulin with different amounts (0–10000 ηg) in milliQ$H_2O$. Fluorescence intensity was measured at 485 nm/535 nm (excitation/emission) wavelengths.

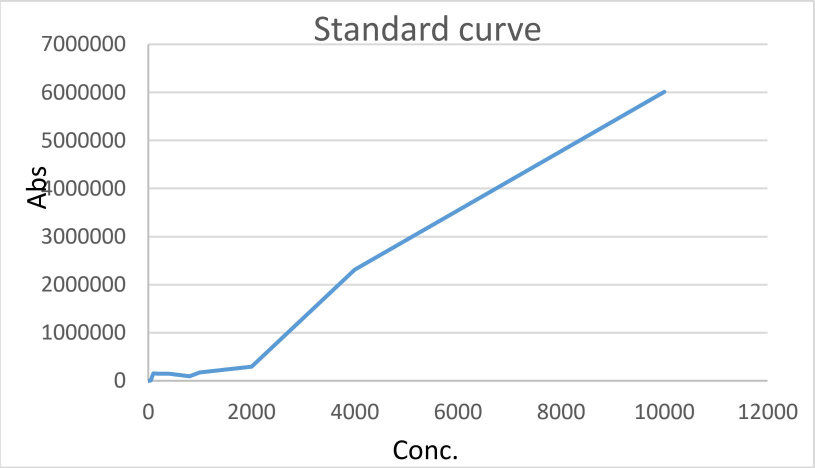


**Figure S1:** Concentration vs. absorbance standard curve prepared with free FITC-Insulin samples. FITC-Insulin was diluted in milliQ$H_2O$. Fluorescence intensity was measured at 485 nm/535 nm (excitation/emission) wavelengths.

## Albumin Loading Efficiency of Ba Salt Particles and Subsequent Release of Albumin from the Particles at Acidic Ph

Different amounts of albumin bound to Ba salt particle were separated from the unbound proteins by centrifugation and ran on SDS-PAGE to see the band intensity of the bound proteins following coomassie dye staining (Supplementary Figure S2). Particles were synthesized in presence of albumin at pH7.8. A few assays were done with albumin-loaded particles following exposure of the particles to harsh acidic pH (pH 1.8). For all 3 types of Ba salt particles ($BaSO_4$, $BaSO_3$ and $BaCO_3$) prepared in presence of 1000 µg/mL, 800 µg/mL and 500 µg/mL of albumin at pH 7.8, the band intensity for the loaded albumin was the same. Albumin band for concentration of 100 µg/mL initially added to prepare the albumin-loaded particles showed slightly lower intensity. That indicates that albumin at 500 µg/mL might saturate the (albumin) binding sites of the particle.

At acidic pH, albumin loaded into $BaSO_4$ particles showed slightly decreased intensity compared to the synthesis pH of 7.8) with clear and intact band. That indicated $BaSO_4$ particle can protect albumin from enzymatic degradation even at very harsh acidic pH. However, albumin loaded to $BaSO_3$ and $BaCO_3$ particles showed significant degradation at harsh acidic pH. For both types of particles, an intact but very low in intensity band was found for highest concentration of 1000 µg/mL of albumin. Bands in lower concentrations of albumin were either too dim in intensity or invisible, indicating significant amount of protein loss even in particle bound form.

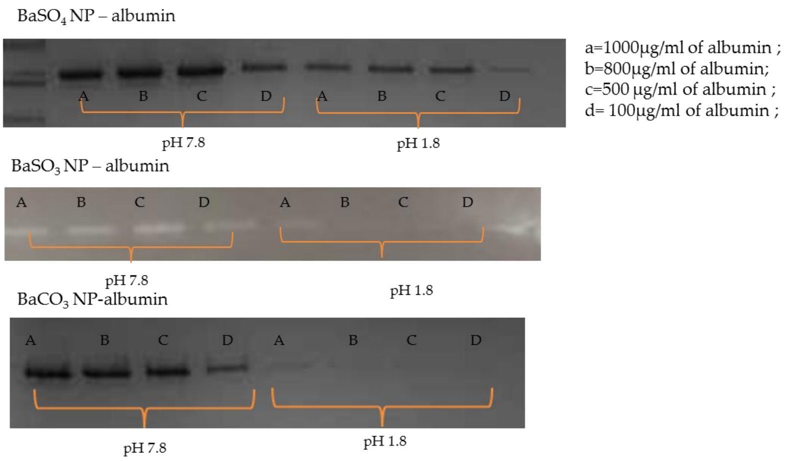


**Figure S2.** Assessment of albumin loading efficiency of Ba salt particle and their ability to protect the protein molecules from an acidic pH (pH 1.8).

Different amounts of albumin (1000 µg/mL, 800 µg/mL and 500 µg/mL and 100 µg/mL) were incubated along with the reactant salt molecules to form albumin-loaded Ba salt particles at pH 7.8. Particles with bound albumin were later exposed to acidic pH (pH 1.8) to test their ability to protect bound albumin in harsh acidic condition. (**a**) Albumin loaded into $BaSO_4$ particles, (**b**) albumin loaded into $BaSO_3$ particles and (**c**) albumin loaded into $BaCO_3$ particles.

### Protection of Albumin Loaded into Ba salt Particles from Enzymatic (Trypsin) Digestion

To assess the potential ability of Ba salt particles to protect particle-bound proteins from enzymatic digestion in gastrointestinal tract, albumin-loaded Ba salt particle were exposed to trypLE (trypsin mimicking enzyme)-added medium prepared with different pHs (pH 7.4, 6.8 and 1.8). Free undigested and trypsin-digested albumin samples were taken as control. The trypsin-digested free albumin showed partial fragmentation, whereas albumin proteins loaded into $BaSO_4$ and $BaSO_3$ particles remained intact in all 3 different pHs (Supplementary Figure S3a). $BaCO_3$ particle-bound albumin was found intact in alkaline pH (7.8 and 6.8); however, the protein band was not visible at acidic pH of 1.8 (Supplementary Figure.S3a). Supplementary Figure S3b shows comparative graphs with relative % of protein degadation for different Ba salt particles. Band intensity was measured using ImageJ. Relative degradation (%) was calculated from the difference in band intensity between trypLE-digested albumin at pH 7.4 and trypLE-digested albumin at subsequent pHs.

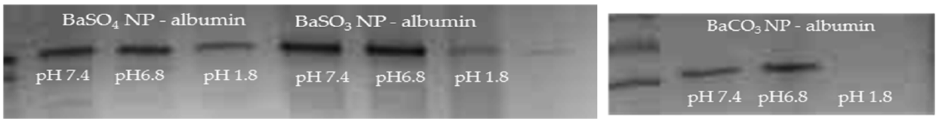


**(a)**

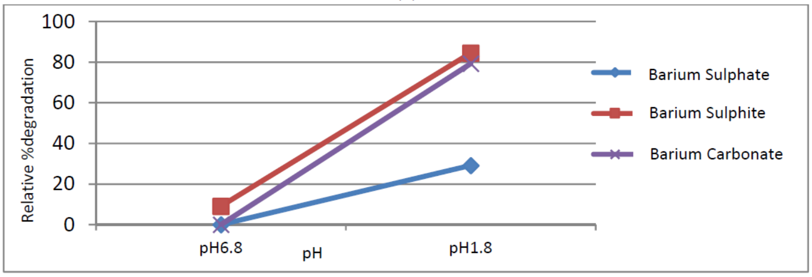


**(b)**

**Figure S3:** Assessment of resistance of albumin loaded into Ba salt particle against enzymatic digestion. Albumin- loaded particles were exposed to trypLE in medium with different pHs (pH 7.4, 6.8 and 1.8). Undigested and trypLE- digested free albumin proteins were taken as control. (**a**) Show the albumin band pictures. (**b**) Shows relative % degradation calculated from band intensities.

## Protection of Albumin Loaded into Ba particle in Simulated Gastric Fluid (sGF)

Particles with loaded albumin were exposed to simulated gastric fluid (sGF). sGF was prepared with added pepsin and in 3 different pHs 5.0, 2.5 and 1, mimicking fluctuating pH of stomach. So the effect observed on band intensity actually reflects the combined effect of enzymatic activity of pepsin and pH. Supplementary Figure S4a shows albumin bands from free albumin without any treatment, followed by 3 rows with free albumin exposed to sGF. No visible band for albumin was found when free albumin was exposed to sGF, whereas the 3 visible bands on gel were from pepsin itself. Albumin when loaded into $BaSO_4$ particle and exposed to sGF with different pHs showed a reduction in band intensity with more acidic pH. Both albumin and pepsin bands were visible at pH 5.0 and pH 2.5. While pepsin band intensity remained the same, albumin band intensity seemed to be dimmed at pH 2.5 compared to pH 5. There was no visible band for albumin or pepsin in pH 1.0. As shown in supplementary Figure S4b albumin loaded into $BaSO_3$ particles and subsequently exposed to sGF with pH 5.0 gave its clear band, whereas loaded albumin exposed to sGF with pH 2.5 showed a decrease in band intensity, and no albumin band was visible at pH 1.0. Supplementary Figure S4c demonstrates that the band intensity of the albumin loaded into $BaCO_3$ particles remained the same when exposed to sGF with pH 5 and pH 2.5, while no visible band was found at pH 1.0.

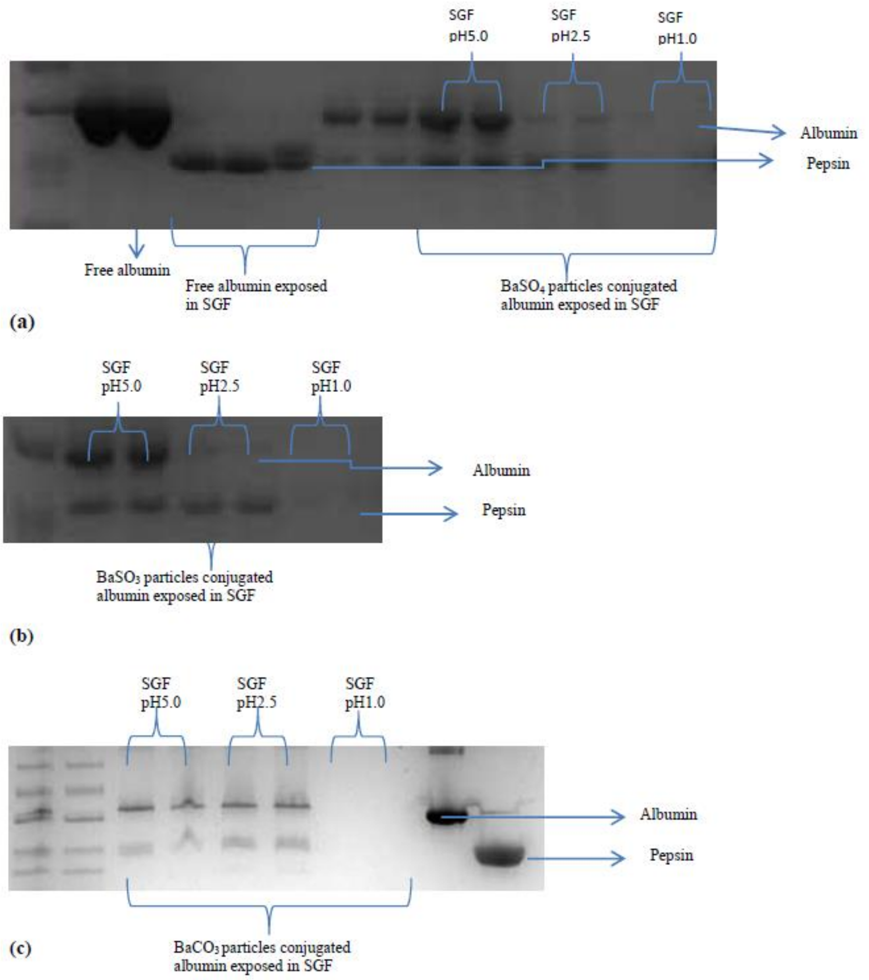


**Figure S4.** Protection of protein loaded into Ba salt particle from enzymatic digestion. Albumin loaded into the particles was exposed to sGF prepared with different pHs (5.0, 2.5 and 1). (**a**) Shows gel picture with free albumin, followed by free albumin exposed to sGF (no visible albumin band), albumin-loaded $BaSO_4$ particles exposed to sGF. (**b**) Shows albumin-loaded $BaSO_3$ particles exposed to sGF and (**c**) shows albumin-loaded $BaCO_3$ particles exposed to sGF.

## Analysis of Effect of Insulin Loading into Particles by FE-SEM

Supplementary Figure S5a shows free $BaSO_4$ particles that are rounder shaped with a distinctive hollow structure at the middle and a smooth outer surface, whereas supplementary Figure S5b demonstrates sample with 2 μg insulin loaded into $BaSO_4$ particles. Low amount of insulin loading showed presence of characteristic free particles along with insulin-loaded particles. Insulin loading changed particle morphology significantly. Insulin-loaded particle were baseball shaped, having a rough surface area without a hollow structure. Supplementary Figure S5c,d show insulin-loaded particles formed in presence of 10 μg and 50 μg of insulin. With an increase in insulin concentration, particles looked more elongated shaped and surface area looked rougher and there were no visible free $BaSO_4$ particle present.

**(a) Particle only**

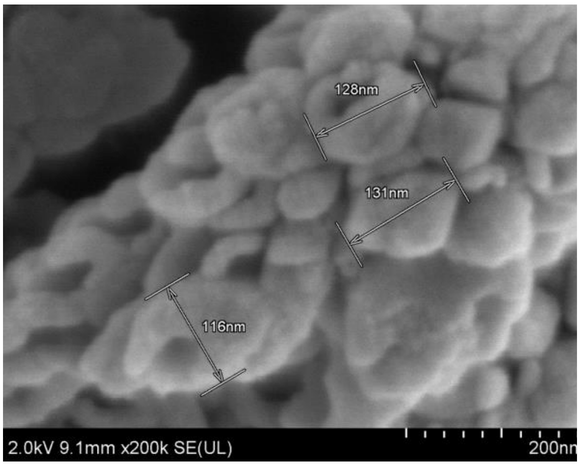


**(b) $BaSO_4$ particle loaded with FITC-Insulin 2 μg**

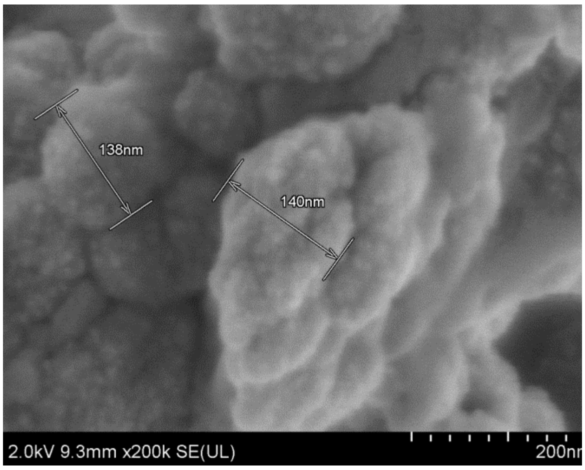


**(c) $BaSO_4$ particle loaded with FITC-Insulin 10 μg**

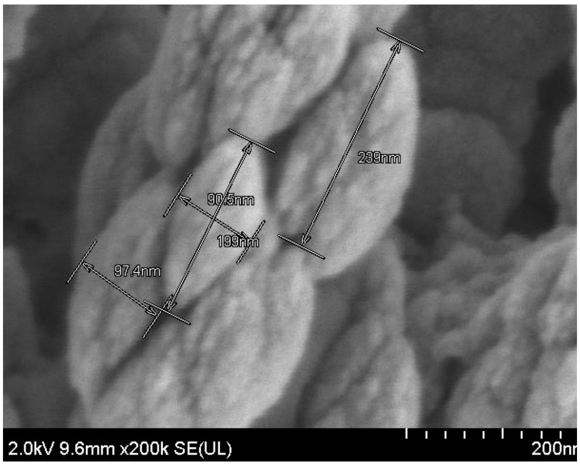


**(d) $BaSO_4$ particle loaded with FITC-Insulin 50 μg**

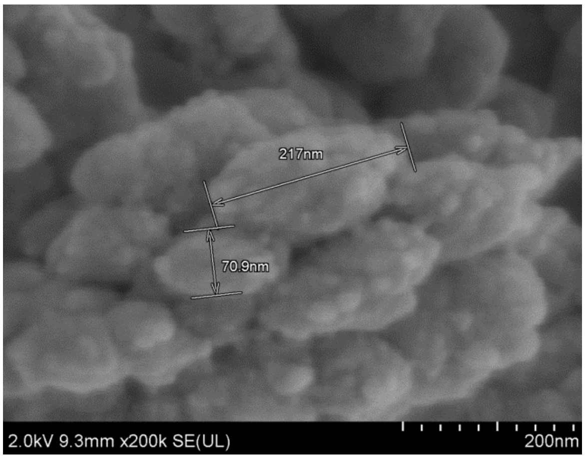


**Figure S5.** FE-SEM images from $BaSO_4$ Particle (free and insulin loaded).Higher magnification pictures at 2.0 kV and 200 k. Different amounts of insulin loading shows different degree of structural changes in particle morphology. (**a**) Free Particle are rounder shaped with a distinctive hollow structure at the middle and smoother outer surface. (**b**) low amount of insulin 2 μg addition to particle show presence of characteristic free particles along with few insulin loaded particle. Insulin loaded particle are baseball shaped, rough surface area and absence of hollow structure. (**c**) and (**d**) shows insulin (10 μg and 50 μg) loaded particle. With increasing amount of insulin, particles look more oval shaped and surface area looks rougher.

Supplementary Figure. S6 shows presence of free and insulin-loaded particle in a single sample when added insulin amount was low (2 μg). Image at the top shows the area in low magnification of 2.0 kV and 50.0 k. Images at the bottom (Figure S6a,b) display free particle (a) and insulin-loaded particle (b) in higher resolution (2.0 kV, 100 k).

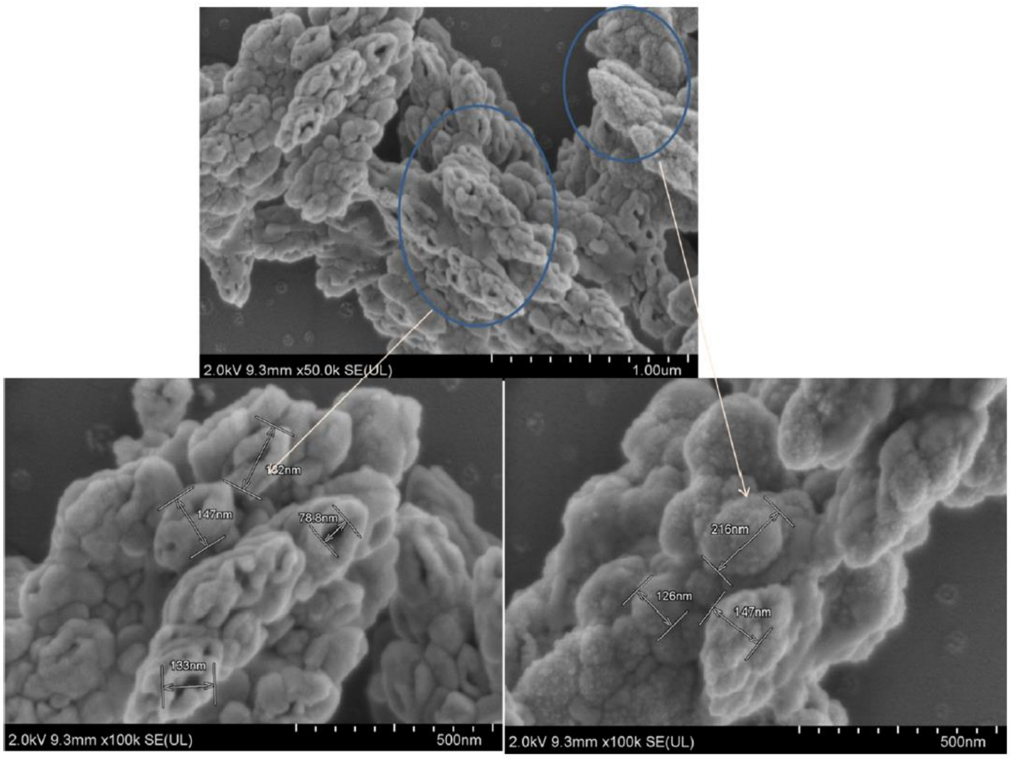


**(a) Free $BaSO_4$ pa** **(b) Insulin loaded BaSO4 particle**

**Figure 6.** FE-SEM images from $BaSO_4$ particle (free and insulin loaded). Free particles are rounder shaped with a distinctive hollow structure at the middle and smoother outer surface. Particles formulated in presence of low amount of insulin (2 μg) resulted in characteristic free particles along with insulin-loaded particles. Insulin-loaded particles are baseball shaped, having a rough surface area without a hollow structure. Image at the top shows presence of both free and insulin-loaded particles in a single sample (2.0 kV and 50.0 k). Free and insulin-loaded particles are identified by distinctive surface morphology. At the bottom, (**a**) shows free particle and (**b**) shows insulin-loaded particle in higher resolution (2.0 kV, 100 k).
